# BMC Biotechnology reviewer acknowledgement 2014

**DOI:** 10.1186/s12896-015-0119-y

**Published:** 2015-02-19

**Authors:** Dafne Solera

**Affiliations:** BioMed Central, Floor 6, 236 Gray’s Inn Road, London, WC1X 8HB UK

## Abstract

The editors of BMC Biotechnology would like to thank all our reviewers who have contributed to the journal in Volume 14 (2014).

**D. Wade Abbott**

Canada

**Karolina Aberg**

USA

**Gabriela Almeida**

Portugal

**Sara Amancio**

Portugal

**Thomas Anchordoquy**

USA

**Denis Archambault**

Canada

**Hideyuki Arimitsu**

Japan

**Aglaia Athanassiadou**

Greece

**Tamas Atlasz**

Hungary

**Brian Austin**

USA

**Eduard Ayuso**

France

**Matteo Ballottari**

Italy

**Jo Ann Banks**

USA

**Priya Baraniak**

USA

**Susanne Barth**

Ireland

**Kristin Baumann**

Austria

**Kyoungwhan Back**

South Korea

**Robert Belshaw**

UK

**Per Berglund**

Sweden

**Frank Bernhard**

Germany

**Maurizio Bettiga**

Sweden

**Andriy Bilichak**

Canada

**Lars Mathias Blank**

Germany

**Murray Boase**

New Zealand

**Mariela Bollati-Fogolin**

Uruguay

**Massimiliano Borgogna**

Italy

**Abhijeet Borole**

USA

**Berrin Bozan**

Turkey

**Mike Brisco**

Australia

**Jochen Büchs**

Germany

**Jan Buer**

Germany

**Pradeep Burma**

India

**John Burnett**

USA

**Azeem Butt**

Pakistan

**Cecília Calado**

Portugal

**Maria Cammareri**

Italy

**Lixiang Cao**

China

**Yongchang Cao**

China

**Sheng-Hao Chao**

Singapore

**Bhushan Chaudhari**

India

**Guo-Qiang Chen**

China

**Sanfeng Chen**

China

**Shyan-Song Chiou**

Taiwan

**Raymond Chollet**

USA

**Yi Chu**

USA

**Dennis Claessen**

Netherlands

**Patrick Coates**

Australia

**Michele Colonna**

Italy

**Gregory Cote**

USA

**Rosymar Coutinho De Lucas**

Brazil

**Don Cowan South**

Africa

**Monika Cserjan**

Austria

**Richard Cummings**

USA

**Rosa M. Cusido**

Spain

**Andre Damasio**

Brazil

**Henri Darmency**

France

**Kasturi Dasgupta**

USA

**Shiladitya Dassarma**

USA

**Lisandra De Castro Bras**

USA

**Ario De Marco**

Slovenia

**Elena Deineko**

Russian Federation

**Zujun Deng**

China

**Ratmir Derda**

Canada

**Sheng-Li Ding**

USA

**Yu Ding**

China

**Ron Dirks**

Netherlands

**Philip Dix**

Ireland

**Jozsef Dobo**

Hungary

**Christian Dölle**

Norway

**Jinhua Dong**

Japan

**Xiaoling Dong**

China

**Veeramuthu Duraipandiyan**

Saudi Arabia

**Anja Ehrhardt**

Germany

**Svetlana Ermolaeva**

Russian Federation

**Jeanni Fehrsen**

South Africa

**Yunjiang Feng**

Australia

**Paulo Fernandes**

Portugal

**Frederico Ferreira**

Portugal

**Kevin Ferreri**

USA

**Paola Foladori**

Italy

**Diogo Fonseca-Pereira**

Portugal

**Bedon Frank**

Australia

**Maria Frantzi**

Germany

**Melissa Fullwood**

Singapore

**Bruria Funkenstein**

Israel

**Sushama Gaikwad**

India

**Junping Gao**

China

**Manuel Garrido**

Portugal

**Brigitte Gasser**

Austria

**Elena Geiser**

Germany

**Richard Gemeinhart**

USA

**Andrea Germer**

Germany

**Ellen Goldman**

USA

**Sailaja Gopalakrishnanchettiar Sivakamiammal**

USA

**Billie Gould**

Canada

**Sibylle Grad**

Switzerland

**Mario Graos**

Portugal

**Rita Graze**

USA

**Teresa Cristina Grisi**

Brazil

**Man Bock Gu**

South Korea

**Santiago Gutierrez**

Spain

**Matthias Hackl**

Austria

**Donny Hanjaya-Putra**

USA

**Michiel Harmsen**

Netherlands

**Colin Harwood**

UK

**Rajni Hatti Kaul**

Sweden

**Dimitris Hatzinikolaou**

Greece

**Klaas Jan Hellingwerf**

Netherlands

**Taira Hidaka**

Japan

**Maria Eugenia Hidalgo Lara**

Mexico

**Thomas Higgins**

Australia

**Nobutaka Hirano**

Japan

**Corinne Hoesli**

Canada

**Dirk Hoffmeister**

Germany

**Mads Vilhelm Hollegaard**

Denmark

**Hyo Jeong Hong**

South Korea

**Danny Hooftman**

UK

**Chienjin Huang**

Taiwan

**Zhaohui Huang**

China

**Matt Hutchings**

UK

**Ee Taek Hwang**

South Korea

**Hafiz Muhammad Nasir Iqbal**

Pakistan

**Tomohiro Ishikawa**

Japan

**Medicharla V Jagannadham**

India

**Sharmili Jagtap**

India

**Grzegorz Janusz**

Poland

**Su Jianguang**

China

**Jonathan Jones**

UK

**Leif J Jonsson**

Sweden

**Franziska Jönsson**

Germany

**Christian Jungreuthmayer**

Austria

**Eszter Kapusi**

Austria

**Levente Karaffa**

Hungary

**Georgios Kararigas**

Germany

**Tadas Kasputis**

USA

**Tatsuya Kato**

Japan

**Rajinder Kaul**

USA

**Yoshinori Kawabe**

Japan

**Arvind Kayastha**

India

**Nawazi Khan**

USA

**Muhammad Azmat Ullah Khan**

USA

**Jin Kim**

USA

**Yeon-Gu Kim**

South Korea

**Nobutada Kimura**

Japan

**David Kirk**

USA

**Hiroshi Kitagaki**

Japan

**Dieter Klemm**

Germany

**Heath Klock**

USA

**Harald Kolmar**

Germany

**Bhupendra Koul**

India

**Andrew Kralicek**

New Zealand

**Florian Krammer**

USA

**Harald Kranz**

Germany

**Anja Kuenz**

Germany

**Wilfried Kues**

Germany

**Peter Kuhnert**

Switzerland

**Prakash Kumar**

Singapore

**Jochen Kumlehn**

Germany

**Masahiro Kurakake**

Japan

**Sunil Kurian**

USA

**Sang-Soo Kwak**

South Korea

**Ying Lai**

USA

**Marcelo Lancellotti**

Brazil

**Ivan Lavandera**

Spain

**Gerald Leblanc**

USA

**Bruno Lefebvre**

France

**Jean-Luc Lenormand**

France

**Thierry Leroy**

France

**Randolph Lewis**

USA

**Dan Li**

China

**Zhimin Li**

China

**Lijia Li**

China

**Ruifang Li**

China

**Gang Li**

China

**Marc Libault**

USA

**Fei-Hu Liu**

China

**Ji-Kai Liu**

China

**Xiudong Liu**

China

**Yiwei Liu**

USA

**Jian-Zhong Liu**

China

**Wusheng Liu**

USA

**Patricia Liwang**

USA

**Agnieszka Loboda**

Poland

**Wayne Loescher**

USA

**Catarina Madeira**

Portugal

**Indu Maiti**

USA

**Miia Mäkelä**

Finland

**Angel Manteca**

Spain

**Angel Maquieira**

Spain

**Eric Marechal**

France

**Alexandra P. Marques**

Portugal

**Seamus Martin**

Ireland

**Ludmila Martinkova**

Czech Republic

**Carla Marusic**

Italy

**Kazuya Matsumoto**

Japan

**Katsuhisa Matsuura**

Japan

**Autar Mattoo**

USA

**Sathiyamoorthy Meiyalaghan**

New Zealand

**Wenzhao Meng**

USA

**Diana Molino**

France

**Lluis Montoliu**

Spain

**Ed Morgan**

New Zealand

**Susann Müller**

Germany

**Ewen Mullins**

Ireland

**Hideo Nakano**

Japan

**Abhay Narayan Singh**

UK

**Diana Nascimento**

Portugal

**Helena Nevalainen**

Australia

**Pablo Ivan Nikel**

Spain

**Benjamin Manuel Nitsche**

Germany

**Kenneth Noll**

USA

**Mohd Nor Norazmi**

Malaysia

**Santosh Noronha**

India

**Cory Nykiforuk**

Canada

**Neftali Ochoa-Alejo**

Mexico

**Marco Oldiges**

Germany

**Berit Olsen Krogh**

Denmark

**Bradley Olson**

USA

**Ron Ophir**

Israel

**Diego Orzaez**

Spain

**Sriram Padmanabhan**

India

**Alok Pandey**

USA

**Soumya Pandit**

India

**Hongbo Pang**

USA

**Patchareewan Pannangpetch**

Thailand

**Francesca Paradisi**

Ireland

**Ashok Patel**

India

**Stephanie Pearl**

USA

**Michael Pearson**

New Zealand

**Nels Pederson**

USA

**Cristina Peixoto**

Portugal

**William Plaxton**

Canada

**Suresh Poda**

USA

**Haryoung Poo**

South Korea

**Rachel Poretsky**

USA

**Thomas Proft**

New Zealand

**Holger Puchta**

Germany

**Francoise Quignard**

France

**Arthur F.J. Ram**

Netherlands

**Sana Raouche**

France

**Mohammad Javad Rasaee**

Iran

**Peter Reilly**

USA

**Craig Richael**

USA

**Maria Manuela Rigano**

Italy

**Ana Filipa Rodrigues**

Portugal

**Maria Elisa Rodrigues**

Portugal

**Gabriela Rodrigues**

Portugal

**Alessandra Rogato**

Italy

**Fernando Rojo**

Spain

**M. Blanca Roncero**

Spain

**German Rosano**

Argentina

**Daniele Rosellini**

Italy

**Joy Roy**

India

**Scott Rudge**

USA

**Edward Rybicki**

South Africa

**Markus Sack**

Germany

**Xavier Saelens**

Belgium

**Debendra K. Sahoo**

India

**Kulvinder Saini**

Saudi Arabia

**Todd Sandrin**

USA

**Vitor Santo**

Portugal

**Nandini Sarkar**

India

**Pooja Saxena**

USA

**Joachim Schiemann**

Germany

**Monica Schmidt**

USA

**Pierre Schneeberger**

Switzerland

**Stefan Scholz**

Germany

**Richard Scott**

New Zealand

**Laura Segatori**

USA

**Junichi Sekiguchi**

Japan

**Davide Seruggia**

Spain

**Ole-Morten Seternes**

Norway

**Jian Shi**

USA

**Zhongping Shi**

China

**Kazuyuki Shimizu**

Japan

**Noriaki Shimizu**

Japan

**Rakesh Shukla**

India

**Sudhir Singh**

India

**Vanga Siva Reddy**

India

**Tobias Sjöblom**

Sweden

**Christopher Mark Smales**

UK

**Allison Snow**

USA

**Ulrich Soltmann**

Germany

**Ruth Elena Soria-Guerra**

Mexico

**Irma Soria-Mercado**

Mexico

**Oliver Spadiut**

Austria

**Rajan Sriraman**

Germany

**Vibha Srivastava**

USA

**Ralf Steuer**

Germany

**Eva Stoger**

Austria

**Krisztina Szabadfi**

Hungary

**Rouzbeh Taghizadeh**

USA

**Fuyuhiko Tamanoi**

USA

**Laszlo Tamas**

Hungary

**Sheng-Ce Tao**

China

**Maureen Taylor**

UK

**Matthew David Templeton**

New Zealand

**Eckhard Thines**

Germany

**Yigang Tong**

China

**Jorg Tost**

France

**Pei-Lan Tsou**

USA

**Maobing Tu**

USA

**Satyanarayana Tulasi**

India

**Kiminori Ushida**

Japan

**Geertje Van Keulen**

UK

**Tom Van Wezel**

Netherlands

**Claudio Varotto**

Italy

**David Vaudry**

France

**Phanindra Velisetty**

USA

**Joachim Venus**

Germany

**Erik Vijgenboom**

Netherlands

**Eva Vincze**

Denmark

**Hans Visser**

Netherlands

**Antonia Vlahou**

Greece

**Moritz Von Stosch**

Portugal

**Masaru Wada**

Japan

**Ronny Wahlström**

Finland

**Kerstin Walter**

Germany

**Guoshun Wang**

USA

**Jiangxin Wang**

China

**Hao Wang**

China

**Yimeng Wang**

USA

**Yihai Wang**

USA

**Yun Wang**

USA

**Heribert Warzecha**

Germany

**Tatsuo Watanabe**

Japan

**Grzegorz Wegrzyn**

Poland

**Ziming Weng**

USA

**Nick Wierckx**

Germany

**Dominic Wong**

USA

**Gavin Wright**

UK

**Jia Xie**

USA

**Danke Xu**

China

**Xingfu Xu**

USA

**Zhenbo Xu**

China

**Montarop Yamabhai**

Thailand

**Boxu Yan**

USA

**William Yang**

USA

**Shinichi Yano**

Japan

**Weijuan Yao**

China

**Jaeseung Yoon**

South Korea

**Jianlan You**

USA

**Jamey Young**

USA

**Xiaobo Yu**

USA

**Qing-Yin Zeng**

China

**Bo Zhang**

USA

**Feng Zhang**

USA

**Shijun Zhang**

USA

**Wenli Zhang**

Germany

**Wei Zhang**

Canada

**Yongxin Zhao**

Canada

**Liang Zhao**

USA

**Fanfan Zhou**

Australia

**Huihao Zhou**

USA

**Jonathan Zuckerman**

USA

